# Inter-arm blood pressure difference and cardiovascular risk estimation in primary care: a pilot study

**DOI:** 10.3399/BJGPO.2021.0242

**Published:** 2022-07-27

**Authors:** Sinead T J McDonagh, Ben Norris, A Jayne Fordham, Maria R Greenwood, Suzanne H Richards, John L Campbell, Christopher E Clark

**Affiliations:** 1 Primary Care Research Group, Institute of Health Services Research, University of Exeter Medical School, College of Medicine & Health, Exeter, UK; 2 Amicus Health - Clare House Surgery, Tiverton, UK; 3 Mid Devon Medical Practice, Witheridge Medical Centre, Tiverton, UK; 4 Leeds Institute of Health Sciences, School of Medicine, University of Leeds, Leeds, UK

**Keywords:** blood pressure determination, hypertension, heart disease risk factors, primary health care, mass screening, general practitioners

## Abstract

**Background:**

Systolic inter-arm differences (IAD) in blood pressure (BP) contribute independently to cardiovascular risk estimates. This can be used to refine predicted risk and guide personalised interventions.

**Aim:**

To model the effect of accounting for IAD in cardiovascular risk estimation in a primary care population free of pre-existing cardiovascular disease.

**Design & setting:**

A cross-sectional analysis of people aged 40–75 years attending NHS Health Checks in one general practice in England.

**Method:**

Simultaneous bilateral BP measurements were made during health checks. QRISK2, atherosclerotic cardiovascular disease (ASCVD), and Framingham cardiovascular risk scores were calculated before and after adjustment for IAD using previously published hazard ratios. Reclassification across guideline-recommended intervention thresholds was analysed.

**Results:**

Data for 334 participants were analysed. Mean (standard deviation) QRISK2, ASCVD, and Framingham scores were 8.0 (6.9), 6.9 (6.5), and 10.7 (8.1), respectively, rising to 8.9 (7.7), 7.1 (6.7), and 11.2 (8.5) after adjustment for IAD. Thirteen (3.9%) participants were reclassified from below to above the 10% QRISK2 threshold, three (0.9%) for the ASCVD 10% threshold, and nine (2.7%) for the Framingham 15% threshold.

**Conclusion:**

Knowledge of IAD can be used to refine cardiovascular risk estimates in primary care. By accounting for IAD, recommendations of interventions for primary prevention of cardiovascular disease can be personalised and treatment offered to those at greater than average risk. When assessing elevated clinic BP readings, both arms should be measured to allow fuller estimation of cardiovascular risk.

## How this fits in

Systolic IADs in BP are independently associated with increased risks of all-cause mortality, cardiovascular mortality, and cardiovascular events. How cardiovascular risk can best be assessed, taking IAD into account, has not been demonstrated. This study applies robust estimates of the additional cardiovascular risk associated with an IAD to a primary care population free of existing vascular disease. The effect of an IAD on reclassification of individuals across commonly used cardiovascular risk intervention thresholds, to refine estimates of risk and personalise treatment decisions, is demonstrated.

## Introduction

Cardiovascular disease is the primary cause of premature morbidity and mortality across the globe, and high BP is a leading contributor to cardiovascular events.^1^ Optimising management of hypertension is therefore recommended by the UK Quality and Outcomes Framework (QOF) for the prevention of cardiovascular disease, and in primary care settings BP measurement is the most frequently undertaken investigation.^2,3^

Typically, individuals with the highest estimated cardiovascular risk gain the most benefit from antihypertensive treatment, but the overall majority of cardiovascular events occur in those at low to medium risk.^4^ Assessment of 10-year cardiovascular risk using established risk scores is a common recommendation of international hypertension guidelines. In the UK, risk assessment for primary prevention of cardiovascular disease is advised by the National Institute for Health and Care Excellence (NICE) using QRISK scores.^5,6^ Similarly the American College of Cardiology/American Heart Association (ACA/AHA) uses ASCVD risk scores based on the ACA/AHA pooled cohort equations,^7,8^ and Hypertension Canada uses the Framingham risk score.^9,10^ Risk scores exceeding defined thresholds are used to guide initiation or intensification of treatment, usually by addition of antihypertensive agents and/or statins. Not all markers of cardiovascular risk identified within guidelines are captured by currently used risk scores. Consideration of such additional markers, indicating possible subclinical arterial disease, could serve to refine and improve selection of people at risk greater than their peers who may, therefore, benefit from more intensive intervention.^11^

Some recognised risk markers for refining intermediate cardiovascular risk, such as assessment of coronary artery calcium, require significant technological investment.^8,12^ Since primary prevention takes place in primary care, any practical identification of additional risk markers should be low cost and feasible for widespread implementation.^13^ Measurement of BP in both arms can feasibly be incorporated into routine primary care without needing additional equipment.^14^ It is recommended internationally in guidelines to accurately assess BP and to determine the higher reading arm for subsequent BP measurement and management. These guidelines also highlight the association of IAD in systolic BP with additional cardiovascular risk.^5,8,10,15^ Despite guideline advice to measure both arms when assessing people for hypertension, this may only be applied in 50% of settings at best.^16^

GP awareness of guideline recommendations is higher than implementation. It has been suggested that presenting clear evidence and justification for recommendations could increase adoption in practice.^17,18^ The authors of the present study have recently reported findings from the large inter-arm BP difference individual participant data (INTERPRESS-IPD) Collaboration, which pooled data from over 53 000 individuals with BP measured in both arms from 24 international cohorts. It confirmed the independent contribution of systolic IAD to cardiovascular risk, and developed and validated risk prediction models that incorporated IAD measurement. It also confirmed and quantified the association of IAD with elevated risk after adjustment for ASCVD, Framingham, or QRISK2 risk scores, providing data that can be directly applied to a primary care population.^19^

The Department of Health introduced the NHS Health Check programme in 2009. It invites individuals aged 40–74 years, who are free of cardiovascular disease, to attend a cardiovascular assessment session, usually in primary care practices, every 5 years.^20^ This session includes BP measurements and other risk marker assessments, thus offering the opportunity to measure BP in both arms to identify IAD in people without a vascular disease diagnosis. The impact of taking account of IAD during cardiovascular risk assessment in a primary care population free of cardiovascular disease has not been demonstrated. Therefore, this pilot study was undertaken to model the application of the authors' adjustments to existing cardiovascular risk scores, taking account of systolic IAD, in a new cohort (not included in the INTERPRESS-IPD Collaboration) presenting to one general practice for routine NHS Health Checks.^19^

## Method

This analysis was undertaken using data collected during the Check-Up study programme.^21,22^

### Participants

From October 2013, patients aged 40–74 years, registered with the Mid Devon Medical Practice (a rural dispensing practice, list size 5000 across three sites in Devon, England), and not already included in any existing vascular disease register, were identified from practice records and invited by letter to book a nurse-led NHS Health Check assessment.^23^ Patients with pre-existing hypertension, atrial fibrillation (AF), CKD, stroke or transient ischaemic attack, heart disease, diabetes, or peripheral arterial disease were excluded.

**Figure 1.**
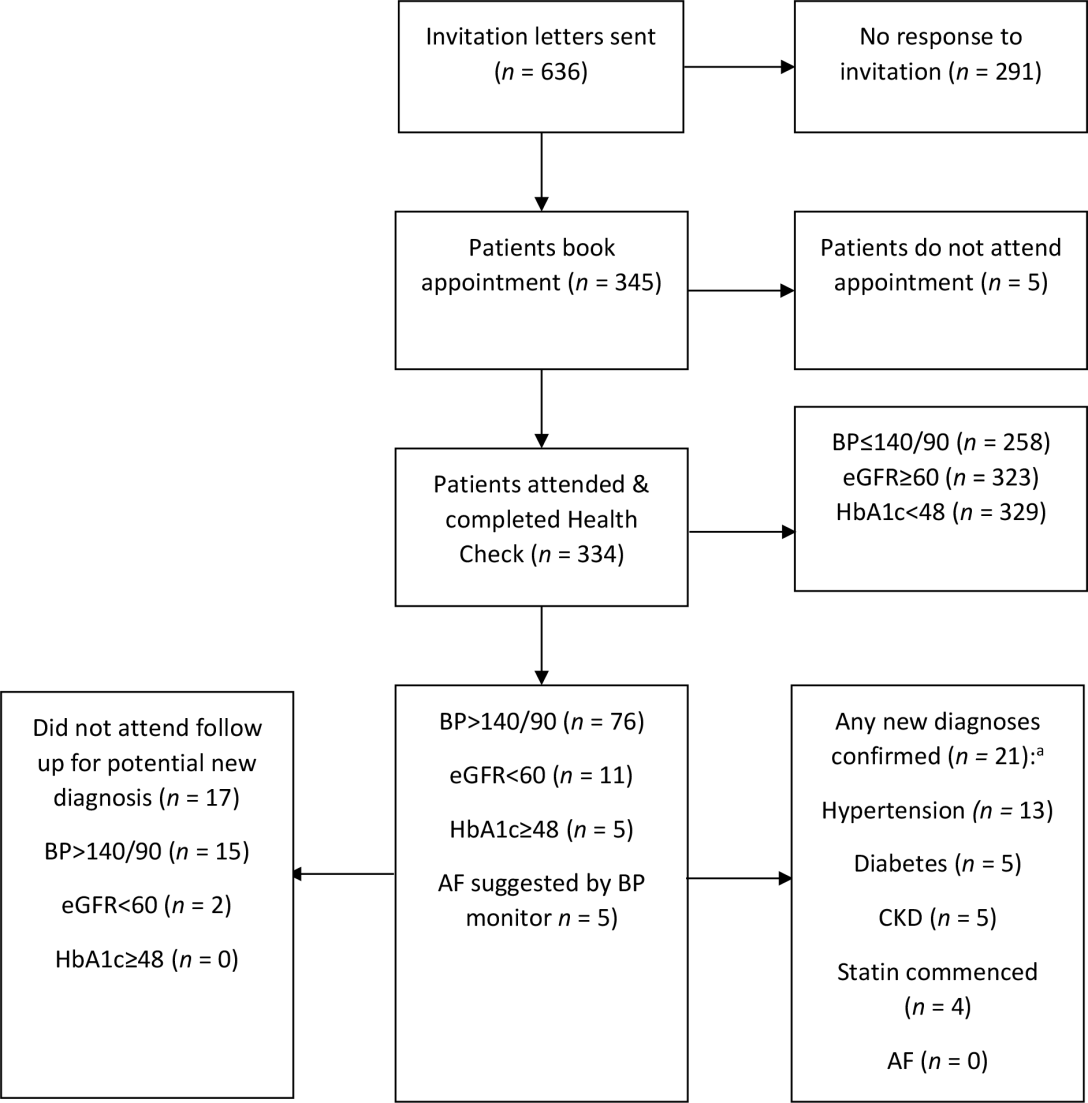
Flow of participants through the health-check protocol during the study. AF = atrial fibrillation. BP = blood pressure. CKD = chronic kidney disease. eGFR = estimated glomerular filtration rate. HbA1c = haemoglobin A1c. ^a^There were 23 new diagnoses in 21 patients.

Patients underwent an NHS Health Check assessment, which included targeted brief health interventions based on lifestyle, history and clinical measurements, and blood sampling. BP was measured using an automated sphygmomanometer (Microlife WatchBP Office, Microlife AG, Switzerland) after 5 minutes of seated rest. This two-cuff device measures three consecutive readings taken one minute apart, simultaneously in both arms, and reports the mean of three readings for each arm. Irregular pulse is also reported; diagnostic electrocardiograms (ECGs) were performed when an irregular pulse was flagged by the device (Figure 1).

Patients were referred for ambulatory blood pressure monitoring if a diagnosis of hypertension was suspected from a clinic reading >140/90 mmHg, and for repeat blood tests if diabetes or CKD were suspected based on the initial investigations (Figure 1). All patients attending the health check received a follow-up letter summarising their results, including a QRISK2 10-year cardiovascular risk assessment score, and lifestyle recommendations. Information on dementia was also supplied to those aged >65 years.

This was a pilot study, undertaken to model application of the authors' adjustments to cardiovascular risk scores, in a single practice cohort with documented IAD, therefore no formal sample size estimates were calculated.

### Analysis

Health check data were collated prospectively in an Excel spreadsheet and analysed using Stata (version 17.0). Descriptive data were summarised as means and standard deviations or proportions, as appropriate. QRISK2 scores were calculated online as part of the health check process^6^; Framingham and ASCVD risk scores were calculated in Stata using published algorithms.^7,9^ The higher reading systolic arm BP was used in all cardiovascular risk calculations. Cardiovascular risk scores were adjusted to take account of measured IAD, by applying hazard ratios derived from our INTERPRESS-IPD Collaboration (Supplementary Figures S2 to S4).^19^ Reclassification across key international hypertension guideline thresholds for intervention, according to estimated cardiovascular risk (NICE 2019: QRISK2 10%; ACC/AHA: ASCVD 10%; Hypertension Canada: Framingham 15%), was calculated by comparing risk scores before and after adjustment of scores for IAD.^5,8,10^ Data analysis was restricted to participants attending and completing health checks; no imputations of missing data were undertaken.

## Results

**Figure 2.**
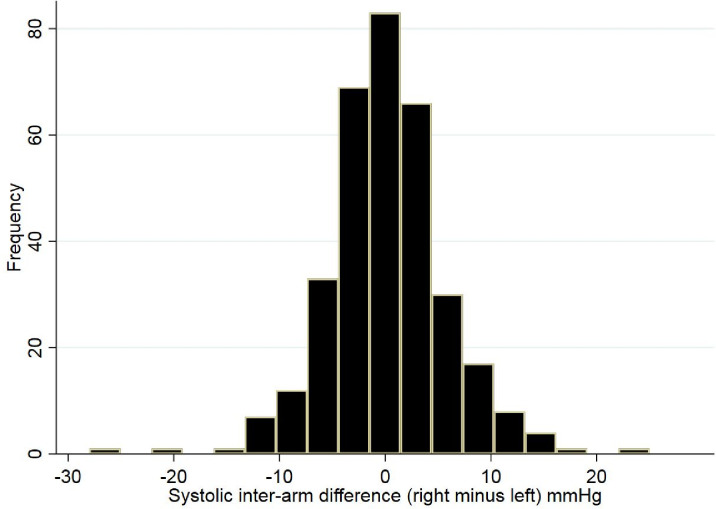
Distribution of systolic inter-arm difference for 334 participants at NHS Health Checks

A total of 1800 patients (36% of registered list) were eligible for invitation to complete an NHS Health Check assessment over the succeeding 5 years from October 2013. Between November 2013 and December 2015, 636 (35%) patients were invited; 340 attended, of whom 334 (53%; 95% confidence interval [CI] = 50% to 57%) attended and completed a health check appointment with full data capture. Mean (standard deviation) age of participants was 57.4 (9.3) years, 58% were female, and mean systolic and diastolic BP was 132 (14)/79 (8.5) mmHg (see Table 1). After appropriate follow-up investigations, new diagnoses of hypertension were confirmed in 13 (3.9%) attenders, type two diabetes mellitus in five (1.5%), and CKD stage 3 in five (1.5%; Figure 1). Of the five (1.5%) participants identified as having an irregular pulse by the WatchBP Office device, none were confirmed to have AF on 12-lead ECGs. Overall, 31 (9.3%) participants had a systolic IAD ≥10 mmHg and 10 (3%) a diastolic IAD ≥10 mmHg at the health check appointment (Figure 2).

**Table 1.** Characteristics of 334 participants at NHS Health Checks

	Mean	SD
Age, years	57.4	9.3
Body mass index, kg/m^2^	26.5	4.5
Alcohol consumption, units/week	8.2	10.2
Systolic blood pressure, mmHg	132.3	13.8
Diastolic blood pressure, mmHg	78.6	8.5
Systolic inter-arm difference, mmHg	0.3	5.7
Absolute systolic inter-arm difference, mmHg	4.2	3.9
Absolute diastolic inter-arm difference, mmHg	3.0	3.4
Total cholesterol, mmol/l	5.7	2.2
HDL cholesterol, mmol/l	1.7	1.1
Glycosylated haemoglobin, mmol/mol	39.1	5.1
Creatinine, mmol/l	78.7	14.7
eGFR, ml/min/1.73 m^2^	80.1	10.3
	***n***	**%**
Female	194	58
Male	140	42
Currently smokes	29	8.7

eGFR = estimated glomerular filtration rate. HDL = high-density lipoprotein.

**Figure 3.**
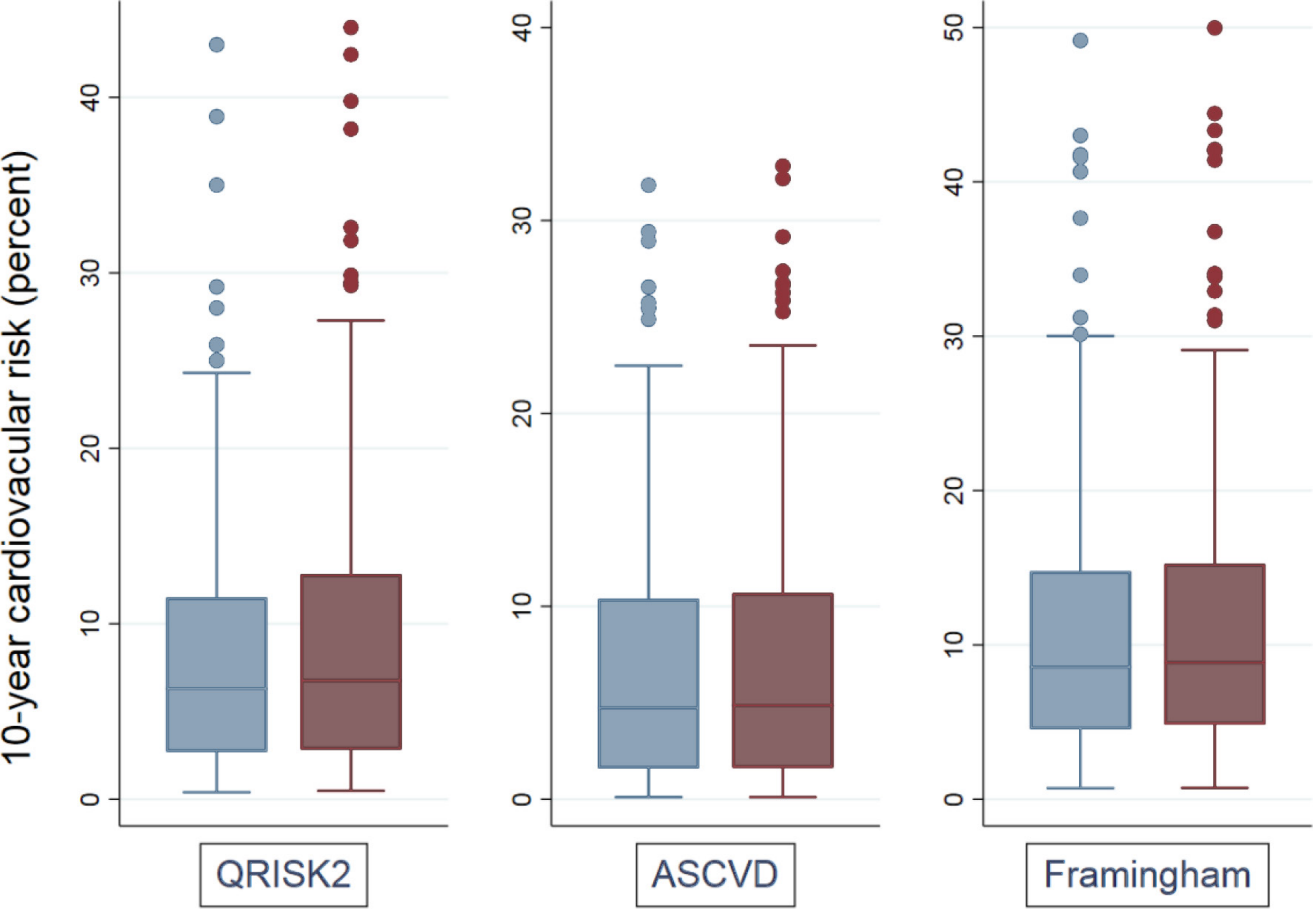
10-year cardiovascular risk scores before (blue, lighter colour) and after (red, darker colour) adjustment for systolic inter-arm difference

Ten-year risks of cardiovascular events, when adjusted for IAD risk, were significantly higher for QRISK2, ASCVD, and Framingham risk scores (*P*<0.001 for all scores; Table 2; Figure 3). By adjusting cardiovascular risk scores to take account of systolic IAD, 13 (3.9%) participants were reclassified from below to above a 10% QRISK2-based treatment threshold; three participants (0.9%) were reclassified from below to above the 10% ASCVD treatment threshold. These represent 13/35 (37%) of participants presenting with an unadjusted QRISK2 between 8% and 9.9%, and 3/29 (10.3%) with an unadjusted ASCVD risk of 8% to 9.9%. For the Framingham 15% intervention threshold, nine (2.7%) were reclassified from below to above the threshold, representing 9/38 (23.7%) of participants with an unadjusted Framingham score between 12% and 14.9% (Table 2; Figure 3)

**Table 2.** Distribution of 10-year cardiovascular risk scores for 334 participants at NHS Health Checks before and after adjustment for systolic inter-arm blood pressure difference

Risk score	Before adjustment for systolic inter-arm difference		After adjustment for systolic inter-arm difference		Differences			
	No (%) above risk intervention threshold^a^	Mean (SD)	No (%) above risk intervention threshold^a^	Adjusted mean (SD)	No (%) reclassified above threshold^b^	*P* value (χ^2^)	Mean difference (SD)^b^	*P* value (*t*-test)
QRISK2	104 (31.1)	8.0 (6.9)	117 (35.0)	8.9 (7.7)	13 (3.9)	<0.001	0.8 (1.4)	<0.001
ASCVD	89 (26.6)	6.9 (6.5)	92 (27.5)	7.1 (6.7)	3 (0.9)	<0.001	0.3 (0.5)	<0.001
Framingham	78 (23.4)	10.7 (8.1)	87 (26.0)	11.2 (8.5)	9 (2.7)	<0.001	0.4 (0.7)	<0.001

^a^Intervention threshold is 10-year cardiovascular risk ≥10% for QRISK2 and ASCVD, ≥15% for Framingham. ^b^Row proportions and means do not always total due to rounding.

No = number.

## Discussion

### Summary

This pilot study has demonstrated that BP can be measured in both arms to determine IAD during routine NHS Health Check assessments. Adjustment of QRISK2 estimated cardiovascular risk to account for IAD reclassified 4% of participants from below to above the NICE QRISK2 intervention threshold. Similar effects were seen when IAD was used to adjust other cardiovascular risk scores. These adjustments are most relevant to those participants with risk scores below but close to intervention thresholds, where adjustment for IAD had a proportionally larger effect on reclassification across risk categories.

These pilot findings have shown that cardiovascular risk classification can usefully be refined, by measuring BP in both arms and taking account of the IAD, during NHS Health Checks.

### Strengths and limitations

Systematic data collection throughout the health check process achieved low levels of missing data. When compared with attenders, non-attenders in this study had twice the smoking rate and significantly higher BP readings in previous primary care records.^22^ Attendance at NHS Health Checks is associated with more positive and proactive attitudes toward personal health care; lower attendance is also observed from people living with higher levels of deprivation.^21,24^ For these reasons, the mean cardiovascular risk for the cohort studied here is likely to be lower than that for the full eligible practice population. These findings are derived from a single rural practice in Devon with low ethnic diversity. Cardiovascular risk varies with ethnic group, but no variation of IAD in England according to ethnicity has been previously shown by the authors.^5,25^ Nevertheless, caution is needed when attempting to generalise the findings to a wider primary care population. The results of this pilot study do, however, illustrate the practical application of the published risk tables (available at: https://medicine.exeter.ac.uk/research/healthresearch/primarycare/interpress-ipd/riskadjustmenttables/) in a primary care setting, indicating the potential use of IAD to refine cardiovascular risk assessment.^19^ The hazard ratios applied to IAD are derived from the separate INTERPRESS-IPD Collaboration; the largest international dataset assembled to examine the implications of an IAD for prediction of mortality and cardiovascular mortality.^19^

This study used the Microlife WatchBP Office device, which is a two-cuff device capable of repeated simultaneous measures with good reproducibility.^26^ The INTERPRESS-IPD Collaboration data are derived largely from sequential BP measurements, which generally yield a greater magnitude in IAD than comparable simultaneous measurements.^27,28^ Consequently, this analysis may have produced estimates of the proportions reclassified by taking account of IAD that are conservative in comparison with sequential assessment of BP in routine practice.

### Comparison with existing literature

Arterial stiffening is an early indicator of hypertension-mediated organ damage, such as left ventricular hypertrophy, which is an important marker of adverse prognosis.^29^ Its presence is suggested in people aged >60 years by a widening pulse pressure (systolic minus diastolic BP >60 mmHg) or at any age by an elevated pulse-wave velocity (PWV).^30^ While pulse pressure is easily calculated, measurement of PWV is not practical in routine primary care. There is good evidence to support the association of systolic IAD with increased arterial stiffness; it is correlated with increased PWV and left ventricular wall thickening.^31–33^ Both arterial stiffness and IAD are associated prospectively with higher rates of cardiovascular events, cardiovascular mortality, and all-cause mortality.^15,19,34^ A systolic IAD has an independent prognostic value for mortality and cardiovascular events over and above that predicted by established risk scores, which the authors believe is explained by its value as a non-invasive indicator of subclinical arterial disease.^11,19^ The current analyses apply the estimates of the impact of systolic IAD on cardiovascular risk scores in a pilot study. They demonstrate the likely impact of assessing an IAD on workload, by refining and increasing the proportions attending an NHS Health Check who will require further investigation for diagnosis and potentially management of hypertension and/or elevated cardiovascular risk.

### Implications for research and practice

In the absence of pre-existing vascular disease, intervention with statin and/or BP-lowering treatment is guided by individual assessment of cardiovascular risk. The pilot findings confirm that a systolic IAD can be applied to refine cardiovascular risk estimates in a UK single primary care population. By taking account of systolic IAD, decisions on interventions for primary prevention of cardiovascular disease can be personalised and could facilitate targeting of treatment to those at greater than average cardiovascular disease risk. The large SMART (specific, measurable, achievable, realistic, and timely) study of 7344 participants, followed over a median of 5.9 years, associated increasing systolic IAD with increased risks of vascular events in people without, but not with, pre-existing vascular disease after carefully adjusted analyses, suggesting that consideration of IAD may be most important for people at low to medium cardiovascular risk.^35^ The NHS Health Check programme is delivered to at least 1 million people annually in England, generating 38 000 new diagnoses of hypertension.^36^ The findings presented here suggest that 4% of these people, which is over 1500 per annum, could be reclassified according to their IAD measurement from below to above the 10% QRISK2 threshold for initiation of BP and lipid-lowering treatment. The low conversion rate of elevated clinic BP readings to diagnoses of hypertension, based on ambulatory BP recordings, emphasises the importance of the NICE diagnostic pathway in avoiding overdiagnosis and overtreatment of hypertension.^5^

In this study, BP was measured simultaneously in both arms using a specific double-cuff device. The WatchBP Office device has been shown to have high specificity for AF, resulting in fewer follow-up ECGs being required where AF is not present. However, sensitivity is variable; too few irregular pulses were flagged in the current study to interpret the device’s performance in place of pulse palpation for a population eligible for NHS Health Checks.^37,38^ In primary care, practitioners rarely have access to equipment that can measure both arms simultaneously; they need a practical and simple method of assessment.^16,39^ Sequential measurement of IAD is the most practical way to implement IAD measurement in primary care.^40^ It will usually overestimate the magnitude of IAD in comparison with simultaneous measurements, but has a high negative predictive value for a simultaneous IAD.^27,28,41^ The INTERPRESS-IPD Collaboration and other sequentially measured cohorts have shown the associations of IAD detected by this method with all-cause mortality, cardiovascular mortality, and cardiovascular events.^15,19,42^ The current pilot findings represents a proof of concept but is likely to have underestimated the true effect of sequentially measured IADs on reclassification of risk. Ambulatory monitoring following an initial raised clinic BP reading to diagnose hypertension is cost-saving owing to better targeting of treatment.^43^ Taking account of IAD should direct more people to this diagnostic pathway; however, the economic impact of this is, as yet, unknown. This pilot study will inform further work to validate this approach, using practical sequential methods of measurement in a larger and ethnically diverse population, which is more representative of the range of people seen in UK primary care.

## Data

Under the terms of approval for the Check-Up study, the dataset is not freely available but can be discussed with the corresponding author on reasonable request.
